# Targeting cellular senescence with novel senotherapeutics by design to extend healthspan

**DOI:** 10.1093/geroni/igab046.2564

**Published:** 2021-12-17

**Authors:** Lei Zhang, Brian Hughes, Luise Angelini, Ryan O’Kelly, Matthew Yousefzadeh, Ted Kamenecka, Laura Niedernhofer, Paul Robbins

**Affiliations:** 1 University of Minnesota, Saint Paul, Minnesota, United States; 2 University of Minnesota, Minneapolis, Minnesota, United States; 3 Scripps Florida, Scripps Florida, Florida, United States

## Abstract

Senescent cells accumulate with age in various tissues and organs, leading to the decline in tissue function and deterioration of many age-related diseases and aging. Senolytics have emerged as an effective therapeutic approach to eliminate senescent cells to improve aging phenotypes and associated co-morbidities. Despite their promising potential, only a handful of senolytics have been reported, including a natural flavonoid fisetin discovered by our group. Fisetin has been shown to reduce senescence, suppress age-related pathology, and extend healthspan in aged mice. However, its moderate potency, potential mutagenic risk and poor bioavailability have limited its further clinical applications. By leveraging drug design, medicinal chemistry and high-content imaging analysis, we have successfully optimized the senolytic activity of fisetin, leading to the identification of two improved fisetin senolytic analogs (FAs) with reduced toxicity in non-senescent cells. The improved senolytic activity of these FAs was demonstrated in murine and human senescent cell models as well as in accelerated aging and naturally aged mouse models. The analysis of the senolytic activity of the FAs as well as several other recently identified senolytics, including a senolytic lipid, will be presented.

